# Analysis of Outcomes of the Nutritional Status in Patients Qualified for Aortic Valve Replacement in Comparison to Healthy Elderly

**DOI:** 10.3390/nu10030304

**Published:** 2018-03-05

**Authors:** Edyta Wernio, Dariusz Jagielak, Jolanta Anna Dardzińska, Ewa Aleksandrowicz-Wrona, Jan Rogowski, Agnieszka Gruszecka, Sylwia Małgorzewicz

**Affiliations:** 1 Department of Clinical Nutrition, Medical University of Gdansk, 80-211 Gdańsk, Poland; o_edyta@gumed.edu.pl (E.W.); annadar@gumed.edu.pl (J.A.D.); ewaalek@gumed.edu.pl (E.A.-W.); 2 Clinic of Cardiac and Vascular Surgery, Medical University of Gdansk, 80-211 Gdansk, Poland; darjag@gumed.edu.pl (D.J.); janrog@gumed.edu.pl (J.R.); 3 Department of Radiology Informatics and Statistics, Medical University of Gdansk, 80-211 Gdansk, Poland; agruszecka@gumed.edu.pl

**Keywords:** elderly, aortic stenosis, nutritional status, body composition

## Abstract

Severe aortic stenosis (AS) is associated with the reduction of muscle mass and may be associated with deterioration of nutritional status. Furthermore, malnourished cardiac patients are characterized by a higher risk of postoperative complications and mortality. The aim of this study was the evaluation and comparison of nutritional status, appetite and body composition in older people with severe aortic stenosis before aortic valve replacement and healthy elderly volunteers. One hundred and one patients, aged >65 years old with severe AS were included in the study. Nutritional status was assessed. Body composition was estimated using bioelectrical impedance analysis. Concentrations of albumin, prealbumin, triglycerides, total cholesterol and C-reactive protein were measured, and a complete blood count was done. About 40% of AS patients were at risk of malnutrition. They had decreased hand grip strength and they lost more body mass than the control group. Malnourished AS patients were older, had lower body mass indexes (BMIs) and lower aortic valve areas in comparison to well-nourished patients. Older AS patients, like their peers, show excessive body mass and, at the same time, the features of malnutrition. They have additional factors such as unintentional weight lost and decreased muscle strength which may be associated with worse outcomes.

## 1. Introduction

The cause of aortic stenosis (AS) in Europe is degenerative calcification associated with the aging process [1,2]. According to a Norwegian study, the prevalence of AS amounts to 1% in people aged 60–69 years old and in 10% of those aged ≥80. However, due to the prolonged life expectancy, the number of patients with AS is expected to rise. Currently, the sole effective method of treating this progressive valve disease is surgical aortic valve replacement [2]. Nowadays, patients of advanced age with severe AS qualify for the mentioned surgical procedure, even despite of the higher risk of complications [3,4,5].

The risk of developing aortic stenosis increases with age, in addition to the risks of deterioration of nutritional status and development of geriatric syndromes, such as frailty syndrome. There are many studies that have evaluated the occurrence of the mentioned syndrome in patients with AS. It should be emphasized that frailty syndrome and nutritional status are related but separate conditions, and both should be diagnosed [6,7,8,9].

It has been proved that poor nutritional status has an impact on the development of geriatric syndromes [8]. Furthermore, malnourished cardiac patients are also characterized by a higher risk of postoperative complications and mortality [10,11].

Elderly with severe AS should be considered as being potentially at risk of malnutrition, due to many reasons—those associated with the normal aging process and with comorbidities. Moreover, symptoms related to the obstruction of blood flow across the aortic valve predispose an individual to impaired appetite, decreased physical activity and, as a consequence, fat-free mass depletion. In previous studies, the body mass index and/or short form of MNA (Mini Nutritional Assessment) evaluation have mainly been used to assess nutritional status. There is a lack of more accurate assessments of nutritional status and comparative analyses with healthy elderly.

Thus, the purpose of our research was the evaluation and comparison of nutritional status, appetite and body composition in older people with severe aortic stenosis before aortic valve replacement (AVR) and healthy elderly volunteers.

## 2. Material and Methods

The study group included 101 elective older patients over 65 years old (mean age 72 ± 4.54 years) with severe aortic stenosis who had qualified for aortic valve replacement, with or without other accompanied cardiac surgery. The examined subjects were qualified for a type of operation by a heart surgeon and were hospitalized at the Department of Cardiac and Vascular Surgery. The medical documentation of all participants was analysed. Patients were examined the day before surgery. The University Ethics Committee approval was obtained.

Severe aortic stenosis was recognized based on echocardiography results: aortic valve area <1 cm^2^ or 0.6 cm^2^/m^2^ body surface area, mean gradient >40 mmHg or peak aortic velocity >4.0 m/s). All participants reported cardiac symptoms of advanced aortic valve disease.

### 2.1. Inclusion Criteria

- Aged over 65 years old
- Severe aortic stenosis, qualified for surgery procedure (TAVI or AVR)
- Absence of severe decompensated concomitant disease, such as pulmonary disease, hepatic insufficiency or kidney insufficiency (K/DOQI stage >3)
- Signed informed consent for participation in the study

### 2.2. Exclusion Criteria

- Aged <65 years old,
- Impaired cognitive function,
- Implanted cardioverter defibrillator,
- Presence of a severe disease, like kidney failure, severe chronic obstructive lung disease, liver cirrhosis or a positive history of cancer
- No consent for participation in the study

In Table 1 characteristics of patients with aortic stenosis are presented.

The control group comprised 75 community-dwelling older volunteers (33 men, 42 women, mean age 70.2 ± 4.55 years) who were participants of the University of the Third Age. In the control group, the criteria for inclusion in the study was aged ≥65 years and lack of any elective surgical procedures and the absence of a cardioverter defibrillator. All those who expressed their willingness to participate in the study were obliged to provide a whole medical history and signed informed consent for participation in the study.

All those who did not meet the inclusion criteria as well as elderly with serious diseases (kidney failure, severe chronic obstructive lung disease, liver cirrhosis or with a positive history of cancer) were excluded. 

### 2.3. Comorbid Diseases

Among the elderly with AS, hypertension (94% of women, 90% of men), coronary artery disease (60% of women, 77% of men), diabetes (31% of women, 28% of men) and hypercholesterolemia (23% of women, 25% of men) were the most frequent comorbidities. Furthermore, the following diseases scored in the Charlson Comorbidity Index (CCI) have been reported: chronic heart failure (8% of women, 11% of men), COPD(Chronic Obstuctive Pulmonary Disease) (2% of women, 15% of men), chronic renal disease (12% of women, 13% of men), peptic ulcer disease (4% of women, 4% of men), mild liver disease (2% of women), rheumatoid arthritis (2% of women, 2% of men), peripheral vascular diseases (6% of women, 4% of men), hemiplegia (2% of men), myocardial infarction (2% of women, 4% of men) and stroke (4% of men).

The control group was mostly affected by hypertension (48% of women, 79% of men) and hypercholesterolemia (40% of women, 58% of men).

Only a few of them suffered from diabetes (2% of women and 6% of men), chronic renal diseases (2% of women), COPD (2% of women, 3% of men), coronary artery disease (7% of women, 15% of men), peptic ulcer disease (2% of women, 6% of men), mild liver disease (2% of women, 3% of men) and peripheral vascular diseases (2% of women, 3% of men).

### 2.4. Anthropometry

Body mass (kg) was measured using a mechanical column scale (Seca 711). A telescopic stadiometer (Seca 220) was used to assess height (cm). Shoulder circumference (as half-length of the non-dominant arm) and calf circumference (at the widest point) were evaluated.

In accordance with the Committee on Diet and Health references [12], body mass index (BMI, kg/m^2^) categories for subjects over 65 years old were accepted:

- Low BMI < 24,
- Normal BMI 24–29,
- High BMI > 29.

Hand grip strength (HGS) was assessed using an analog hand-held dynamometer (Baseline 12-0240, Burr Ridge, IL, USA). Norms for HGS according to BMI and gender were adopted from The European Working Group on Sarcopenia in Older People [13]. 

The percent of body mass lost within the last 6 months was also calculated.

For the evaluation of body composition parameters, such as fat free mass (FFM; kg, %), body fat (kg, %), muscle mass (MM; kg) and phase angle for 50 kHz, bioelectrical impedance analysis (BIA) with Maltron BioScan 920-2 (Rayleigh, Essex SS6 7XF, UK) was carried out. BIA was performed in a fasted state, using tetrapolar techniques (hand–foot). The results were used to calculate fat free mass index (FFMI; kg/m^2^) as FFM (kg)/height (m)^2^, Fat mass index (FMI; kg/m^2^) as body fat (kg)/height (m)^2^ and muscle mass index (MMI; kg/m^2^) as MM (kg)/height (m^2^).

### 2.5. Nutritional Status

Nutritional status was assessed with a full version of the Mini Nutritional Assessment (f-MNA) and the 7-point Subjective Global Assessment (7-SGA). According to the f-MNA, subjects were considered to be well-nourished when f-MNA ranged between 24 and 30, at risk of malnutrition when they received 17–23.5 points and malnourished if the score was <17 [14].

The 7-point Subjective Global Assessment (7-SGA) classified patients as well-nourished when they received 6–7 points, moderately/mild malnourished when they received 4–5 points, and severely malnourished when they had 1–3 points [15]. 

The Simplified Nutritional Assessment Questionnaire (SNAQ) was used to evaluate appetite. A result of ≤14 points indicated a decreased appetite and a related risk of body mass loss within the following 6 months [16].

### 2.6. Comorbidity Index

The age-adjusted Charlson Comorbidity Index (CCI) was calculated in accordance with the method published by Charlson et al. [17]. The CCI includes 19 diseases with corresponding weights. Furthermore, the final result contains additional points for each decade after 40 years of age (1 point for ages 41–50, 2 points for ages 51–60, 3 points for ages 61–70 and 4 points for 71 years of age or older) [17].

### 2.7. Biochemistry

Concentrations of albumin, prealbumin, C-reactive protein, lipidogram and complete blood count (WBC, PLT, RBC, Hb) were measured in patients with AS by routine laboratory methods before surgery, during hospitalization. The blood was collected after overnight fasting.

### 2.8. Statistical Analysis

The statistical analysis was performed with Statistica 12.0 for Windows. The Shapiro–Wilk test was used to assess the distribution of variables. The differences between groups were calculated by Student’s *t*-tests or by U Mann–Whitney tests, depending on the distribution. Data is presented as the mean ± standard deviation (SD) or median and range. Analyses of correlations were done using Spearman’s test. The χ^2^ Pearson’s test was also used.

A Logistic regression analysis was performed to evaluate the odds ratio of the deterioration of nutritional status and impaired appetite in the study population. A *p*-value of < 0.05 was considered to be statistically significant.

## 3. Results

### 3.1. Nutritional Status

Considering the full-MNA, 42% (*n* = 42) of patients with AS and 8% (*n* = 6) in the control group were at risk of malnutrition, 2% (*n* = 2) and none in the control group were malnourished. In the gender analysis, relevant differences in f-MNA between AS and the control group were observed (men 25.5 vs. 27.0 points, *p* < 0.001; women 24.0 vs. 27.0 points, *p* < 0.001).

Similarly, results from the 7-SGA showed that 2% (*n* = 2) of older people with AS and none of the healthy elderly were malnourished; moreover, 40% (*n* = 40) of AS subjects and 15% (*n* = 11) of the control group were considered to be mildly/moderately malnourished. As presented in Table 2, differences in the 7-SGA assessment between studied groups were statistically significant. The statistical analysis showed a correlation between f-MNA and 7-SGA (the McNemar’s test, *p* = 0.003).

A decreased appetite (SNAQ score ≤14 points) was detected in 28% of females with AS and in 26% of men with AS (see Table 2). The results of the SNAQ were statistically lower in AS patients in comparison to controls (15.7 vs. 17.0, respectively, *p* < 0.001).

In the AS group, the mean s-albumin level was 36.7 ± 6.7 g/L (range 19.6–49.0) and the mean prealbumin level was 31.83 ± 7.03 mg/dL (range 15.7–49.7). Low albumin levels (<35 g/L) were presented by 41.1% of patients. No statistically significant correlations between biochemical parameters and 7-SGA or f-MNA were observed. The biochemical parameters are presented in Table 3.

### 3.2. Anthropometric and Functional Measurements

The mean BMI in the AS group was 28.9 ± 5.7 (range 19.2–50.2); In comparison, the average BMI of elderly volunteers was statistically (*p* = 0.03) lower: 27.3 ± 3.4 (range 20.3–35.5). In the AS group, 18.5% of patients had a BMI < 24.0, 39.6% had a BMI between 24.0 and 29.0 and 42.5% of patients had a BMI > 29.1. HGS was lower in the AS group than in controls (29.6 ± 9.5 vs. 29.6 ± 9.6; *p* = 0.03).

However, the comparative analysis revealed that men with AS had lower mid-arm and calf circumferences than controls (mid arm: 29 cm vs. 31 cm, *p* = 0.003; calf: 36 cm vs. 38 cm, *p* = 0.001). No differences were noted for BMI or body composition between the male groups.

Women with AS were characterized by a lower percentage of fat-free mass (57.7% vs. 61.9%, *p* = 0.031) and lower phase angle (8.17° vs. 9.71°, *p* = 0.022), as well as a higher BMI (29.6 kg/m^2^ vs. 26.8 kg/m^2^, *p* = 0.014) and fat mass index (12.8 kg/m^2^ vs. 10.4 kg/m^2^, *p* = 0.042) in comparison with healthy women. It was noted that hospitalized AS patients had decreased hand grip strength and they lost, unintentionally, more body mass over the 6-month period than the control group (Table 4).

### 3.3. The Frequency of Nutritional Status Deterioration, Appetite and Muscle Mass Related Parameters in Elderly, with and without Aortic Stenosis

Considering all individuals, in the group of elderly with AS, decreased hand grip strength was more common than in the control group (χ^2^ = 11.57, *df* = 1, *p* < 0.001), as well unintentional weight lost within 6 months (χ^2^ = 22.37, *df* = 1, *p* < 0.001), low phase angle (χ^2^ = 8.15 *df* = 1, *p* = 0.004), impaired appetite (χ^2^ = 11.64, *df* = 1, *p* < 0.001) and poor nutritional status, according to the f-MNA (χ^2^ = 22.47, *df* = 1, *p* < 0.001) and 7-SGA (χ^2^ = 15.76, *df* = 1, *p* < 0.001).

In the group of people ≥75 years old, the frequencies of unintentional weight lost (χ^2^ = 6.04, *df* = 1, *p* = 0.014) decreased hand grip strength (χ^2^ = 9.64, *df* = 1, *p* = 0.002) and poor appetite (χ^2^ = 7.34, *df* = 1, *p* = 0.001) were more common in those with AS. In addition, they presented worse nutritional status’ according to f-MNA (χ^2^ = 9.37, *df* = 1, *p* = 0.002) and 7-SGA (χ^2^ = 3.71, *df* = 1, *p* = 0.049) compared to healthy volunteers over 75 years old.

In the elderly >75 years old with AS, decreased appetite (χ^2^ = 8.65, *df* = 1, *p* = 0.009) and unintentional weight loss (χ^2^ = 12.66, *df* = 1, *p* < 0.001) were diagnosed more frequently in comparison with the control group. 

Malnourished patients with AS were significantly older, with lower BMIs, and less fat mass and muscle mass. However, they had lower appetites, lower AVA and their unintentional body weight loss was significantly higher (mean 2.98%) in comparison to well-nourished patients (mean 0.89%). No differences in serum albumin or prealbumin were observed. The comparison between malnourished and well-nourished AS patients is presented in Table 5.

There were positive correlations between f-MNA results and aortic valve area (cm^2^), and negative correlations between 7-SGA and EUROScore II were observed. In the group of elderly with aortic stenosis, both f-MNA and 7-SGA correlated positively with HGS and anthropometric measurements as well with body composition parameters, and correlated negatively with the Charlson Comorbidity Index, age and percentage of unintentional weight loss (see Table 6). No correlations between biochemical parameters and anthropometry, nor between SGA and f-MNA were noticed.

Results of the logistic regression analysis show that the deterioration of nutritional status, as measured by the 7-SGA and f-MNA, was associated with the presence of aortic stenosis (respectively, OR 3.6 (1.3–10.17) and OR 8.5 (2.89–25.26)), low BMI (OR 13 (4.39–40.35) and OR 6.5 (2.29–18.96)) as well unintentional weight lost (OR 4 (1.7–9.53) and OR 1.9 (1.28–6.6)) (Table 7).

An impaired appetite was related to the presence of aortic stenosis (OR 5.1 (1.85–14.11)) and advanced age increased the risk of poor nutritional status (7-SGA: OR 2.57 (1.12–5.87)).

## 4. Discussion

This study included patients with severe aortic stenosis over 65 years old who had qualified for surgery and elderly volunteers without serious diseases. Symptomatic AS may impact a patient’s appetite and nutritional status, and screening for signs of malnutrition should be an integral part of the assessment of a patient with chronic heart disease, especially before surgical intervention. In our previous study the significant prevalence of malnutrition in this group of patients was described, along with proof that 7-SGA is a reliable tool to assess malnutrition [18]. Any deviations from the norm might negatively impact treatment outcomes. In the presented study, for the first time, we compared nutritional status, appetite and body composition of elderly with severe AS who had qualified for AVR and older people without serious chronic diseases. 

Previous studies suggested a correlation between BMI and nutritional status, and post-operative complications. Authors showed that when BMI was <24 kg/m^2^ the risk of decline in nutritional status was 13-fold higher based on the 7-SGA results and 6.5-fold higher based on the f-MNA results. Therefore, in patients with AS and low BMI simultaneously, the risks related to the surgical procedure may be also associated with poor nutritional status [10,11]. The other studies showed a correlation between low BMI and an increased risk of postoperative complications and mortality after AVR [19,20]. Arsalan et al., demonstrated that the frequency of adverse outcomes after TAVI was greater when the BMI was below 23 kg/m^2^ [19]. Also, Koifman et al. noticed an increased risk of adverse effects when the BMI was lower than 20 kg/m^2^ [20].

On the other hand, in our study group, despite having a normal or elevated BMI, it was found that the majority of patients were at risk of malnutrition, or mild malnutrition criteria were met. The assessment of nutritional status was done comprehensively. Recommended scales for the assessment of nutritional status and biochemical indicators, as mentioned above, were used. It is worth stressing that such a comprehensive assessment is necessary to obtain adequate results, since the measurement of body mass and BMI itself is insufficient and not entirely reliable. According to both–f-MNA and SGA, about 40% of the AS patients were at risk of malnutrition or were malnourished, compared to 6% of the control group.

The unintentional body mass lost in the AS group was significantly higher than in the control group and was associated with a worse nutritional status. What is more, the presence of a decreased appetite (according to the SNAQ) was also more common in AS patients, which is unequivocally connected with the risks of further weight and muscle loss within the following 6 months. The results of the SNAQ showed that hospitalized patients with AS often complained of a lack of appetite or a rapid feeling of fullness. The rating of appetite seems to be especially important, because in most cases, malnutrition may be due to eating inadequate portions and/or meals poor in nutrients [16].

As presented in other studies, unintentional body mass loss is an unfavorable factor in chronically ill patients [21]. Previous research has indicated that the prevalence of malnutrition is high, and about 80% of people over 65 years of age at the beginning of hospitalization presented signs of malnutrition [22]. In patients over 65 years, there is a natural reduction in the content of lean body mass. Furthermore, this group of people is significantly more likely to suffer from diabetes, hypertension, cancer, kidney disease, peptic ulcer disease, and coronary disease, which have significant impacts on the deterioration of nutritional status [22].

No statistical correlations between s-albumin or s-prealbumin and the other nutrition indices were observed in our population. However, it should be underlined that 41% of AS patients presented an albumin concentration below the references value. Higher serum albumin levels are associated with an improved prognosis, fewer complications after surgery, and longer survival time. In some studies, hypoalbuminemia (<3.5 g/dL) was a strong factor associated with a longer hospitalization time and greater complications after surgical management [23]. Discrepancies between biochemical and anthropometrical or clinical results indicate the need for a comprehensive assessment of the patient based on the recommended methods. Usually, changes in body composition precede the reduction of serum albumin and can show the aging of a human, and, on the other hand, are a necessary element for recognizing sarcopenia or frailty syndrome.

In our study, patients classified as malnourished were statistically older (73 vs. 54 years old), with lower BMIs and lower AVAs. Similarly, Tangvik et al., reported that deterioration of nutritional status is associated with age [24]. The influence of severe diseases, such as aortic stenosis, on appetite and the other parameters of nutritional status is certain, but studies examining the state of nutrition in this disease are lacking. A logistic regression analysis showed, in our patients, that aortic stenosis is associated with an impaired appetite. As a consequence of reduced appetite, disease and advanced age, we observed some changes in the body composition of AS patients.

Male patients with AS were characterized by decreased calf and mid-arm circumferences in comparison with control men, which may indicate a reduced level of physical activity [25]. In turn, female patients were characterized by higher body weights and fatness than the control females. Kamiya et al. examined 599 older patients with chronic vascular disease and found that MAC, but not CC, was related to the decreased function of skeletal muscle and showed a significant prognostic capability, independent of other predictors. Nevertheless, a higher CC was associated with a better prognosis [26,27]. 

Our findings revealed that elderly with AS had lower hand grip strengths in comparison to healthy older volunteers, despite no differences in muscle mass content. Moreover, low HGS was diagnosed significantly more often in the group of cardiac patients ≥75 years old, but not in the younger group (<75 years old).

Advanced age, chronic diseases and disability are the main factors affecting the impairment of muscle function. The diagnosis of muscle weakness is part of the criteria for the recognition of frailty syndrome as well as sarcopenia and correlates with nutritional status [6,7,8,13,28,29]. The content of the muscle mass may not reflect muscle function; this has been proven in large cohort studies [26,30]. Decreased HGS is a sign of a lowering in the quality of muscle as part of the aging process; these abnormalities are enhanced by a poor health condition e [28,29,30].

Thus, we found that the risk factors of postoperative complications and mortality occur in the elderly classified with AVR, especially those with decreased hand grip strength and unintentional body weight loss [6,7,8,9,31]. Stortecky et al., demonstrated that 44% of patients qualified to TAVI were at risk of malnutrition; furthermore, the deterioration of nutritional status increase the risk of death one year after the procedure by nearly seven-fold [31]. It has been documented that preoperative unintentional weight loss results in adverse effects on postoperative outcomes [32]. Moreover, decreased body weight is also observed after cardiac surgery and may intensify problems associated with nutritional status and outcomes, even in those with a high BMI [33].

Adequate nutrition affect energy and protein metabolism in the early recovery period after cardiac surgery and may improve patients’ outcomes [34]. We revealed that in those with AS, there is often an impaired appetite, which may lead to unintentional weight lost as well as macro- and micronutrient depletion. All these factors may have an impact on recovery after cardiac surgery and the occurrence of complications.

The limitation of our study is the relatively small group of patients; however, despite this limitation, the results of the present study are valuable because that they indicate preexistent malnutrition in patients with AS and a risk of further deepening malnutrition (SNAQ). It should be taken into account that aortic stenosis, which is often recognized and/or treated surgically in the later stages of life, may—independently from advanced age and connected multimorbidity—affect the appetite and nutritional status and consequently lead to unintentional weight loss and depletion of muscle function. Deterioration of nutritional status may be related to worsening of the disease. Assessing nutritional status in AS patients may provide clinically relevant information, not only in situations where surgical treatment is needed. This is especially important in view of today’s prolonged life expectancy and therefore, presumable increase in the number of patients with AS. The evaluation of nutritional status should become a part of both routine clinical assessment, and prognostic stratification. The clinical benefits of nutritional intervention should be further studied.

## 5. Conclusions

The results of the study indicate preexistent malnutrition in patients with AS, and a risk of further deepening malnutrition. Nutritional status assessment in AS patients may provide clinically relevant information, and it is especially important in view of today’s prolonged life expectancy and therefore, presumable increase in the number of patients with AS. Older people with aortic valve stenosis, like their peers, show excessive body mass and, at the same time, features of malnutrition. However, due to the disease, they have additional factors, such as unintentional weight loss and decreased muscle strength, which may be associated with worse outcomes.

## Figures and Tables

**Table 1:** Characteristics of patients with aortic stenosis. Data are presented as mean ± SD/range.

Parameters	Elderly Men with AS (*N* = 53)	Elderly Women with AS (*N* = 48)
NYHA class I/II/III/IV%	2/40/53/5	2/40/56/2
AVA cm^2^	0.76 ± 0.17	0.7 ± 0.16
Mean gradient mm Hg	46 ± 12	48 ± 14
Peak aortic velocity m/s	4.4 ± 0.6	4.4 ± 0.6
%LVEF (range)	50 (15–65)	50 (35–80)
EURO Score II (range)	1.64 (0.72–72.01)	2.2 (0.93–11.10)
Qualification for a surgical procedure *n* (%)		
AVR	30 (57)	24 (50)
AVR + CABG	13 (24)	19 (40)
AVR + MVR	1 (2)	0
AVR + MVR + CABG	0 (0)	2 (4)
TAVI	9 (17)	3 (6)

AS—aortic stenosis; NYHA—New York Heart Association; AVA—aortic valve area; LVEF—Left Ventricular Ejection Fraction; AVR—aortic valve replacement; CABG—coronary artery bypass graft; TAVI—transcatheter aortic valve implantation; MVR—mitral valve replacement; EUROScore II-European System for Cardiac Operative Risk Evaluation.

**Table 2:** Results of nutritional status and appetite assessment. Data are presented as mean ± SD or range.

Parameters	Men with AS *n* = 53	Men without AS *n* = 33	*p*	Women with AS *n* = 48	Women without AS *n* = 42	*p*
Full-MNA (points)	25.5 (17–29.5)	27 (20.5–29)	<0.00	24 (18–29)	27 (22.5–30)	<0.00
7-SGA (points)	5.4 (3–6)	5.6 (5–6)	0.03	5.4 (3–6)	5.6 (5–6)	0.00
SNAQ (points)	15.8 ± 1.9	16.6 ± 1.6	0.05	15.7 ± 1.7	17.3 ± 1.9	<0.00

AS—aortic stenosis; MNA—Mini Nutritional Assessment; SGA—Subjective Global Assessment; SNAQ—Simplified Nutritional Appetite Questionnaire.

**Table 3:** Biochemical parameters in the AS group.

Parameters	AS Group	References Values
Albumin g/L	36.7 ± 6.7	35–50
Prealbumin mg/dL	31.83 ± 7.03	19.5–35.8
WBC × 10^9^/L	7.6 ± 2.1	4–10
Total number of lymphocyte/1 mm^3^	1.8 ± 0.6	>1.5
RBC × 10^9^/L	4.4 ± 0.5	4.5–5.5
Hb g/dL	13.2 ± 1.4	13–17
Plt × 10^9^/L	218.2 ± 53.4	150–410
CRP mg/dL	3.9 ± 4.9	0–5
Total Cholesterol mg/dL	144.1 ± 42.1	115–190
Triglycerides mg/dL	123.9 ± 128.6	<150

The data is presented as means ± SD; AS—aortic stenosis; WBC—white blood cells; RBC—red blood cells; Hb—hemoglobin; Plt—platelets; CRP—C reactive protein.

**Table 4:** The comparison of anthropometric measurements and body composition parameters in AS and control groups.

Parameters (mean ± SD or range, Min.–Max.)	Men with AS *n* = 53	Men without AS *n* = 33	*p*	Women with AS *n* = 48	Women without AS *n* = 42	*p*
Age adjusted CCI	4 (2–9)	3 (2–5)	<0.00	3 (5–8)	3 (2–5)	<0.00
BMI (kg/m^2^)	28.1 ± 5.2	28 ± 3.7	0.55	29.8 ± 6	26.8 ± 3.2	0.01
CC (cm)	36 (26–42)	38 (31–43)	0.00	35 (28–43)	34 (25–39)	0.10
MAC (cm)	29 (23–39)	31 (25–37.5)	0.00	28 (21–37)	28 (24–32)	0.12
% of WL in 6 months	0 (0–8.9)	0 (0–7)	0.02	0 (0–11.3)	0 (0–2.8)	<0.00
HGS (kg)	33.5 (16–46)	38.0 (29–60)	0.00	20.0 (6–32)	21.5 (13–33)	0.02
FFM (kg)	58.7 ± 8.6	57.9 ± 5.6	0.91	42.1 ± 5.2	42.1 ± 3.4	0.67
FFM (%)	71.4 ± 8	70.7 ± 4.9	0.42	57.7 ± 9.2	61.9 ± 7.5	0.03
FAT (kg)	22.3 (8.1–65.3)	23.4 (10.9–37.5)	0.50	29 (4.0–78.8)	25.5 (7.6–45)	0.08
FAT (%)	28.4 ± 8	29.3 ± 4.8	0.43	42.3 ± 9.2	38 ± 7.5	0.31
MM (kg)	27.5 ± 3.7	27.6 ± 2.7	0.69	17.6 ± 1.9	18 ± 1.2	0.52
Phase angle (°)	8.78 (5.52–14.66)	8.65 (7.96–11.1)	0.78	8.17 (6.25–19.65)	9.71 (7.03–14.17)	0.02
FFMI (kg/m^2^)	19.9 ± 2.5	19.7 ± 1.5	0.96	16.7 ± 1.3	16.4 ± 1	0.05
FMI (kg/m^2^)	8.43 ± 3.9	8.3 ± 2.3	0.49	12.8 ± 5.5	10.4 ± 3.1	0.04
MMI (kg/m^2^)	9.4 ± 1.1	9.7 ± 0.8	0.60	7 ± 0.4	7 ± 0.25	0.94

AS—aortic stenosis; CCI—Charlson comorbidity index; BMI—body mass index; MAC—mid-arm circumference; CC—calf circumference; HGS—hand grip strength; FFM—fat-free mass; FAT—fat mass; FFMI—fat-free mass index; FMI—fat mass index; MMI—muscle mass index; WL—weight loss.

**Table 5:** The comparison between well-nourished and malnourished patients with AS. Data is presented as mean ± SD.

Parameters	Malnourished AS Patients	Well-Nourished AS Patients	*p*
Age (years)	76.14 ± 5.58	73.50 ± 4.78	0.01
HGS (kg)	24.52 ± 9.84	27.75 ± 9.24	0.12
BMI (kg/m^2^)	26.76 ± 5.43	30.46 ± 5.44	0.00
FFMI (kg/m^2^)	17.47 ± 2.31	19.06 ± 2.55	0.00
Muscle Index (kg/m^2^)	7.80 ± 1.30	8.55 ± 1.46	0.00
MAMC (cm)	27.45 ± 3.10	30.44 ± 3.56	0.00
CC (cm)	33.76 ± 3.68	36.47 ± 3.01	0.00
% of lost body mass	2.92 ± 3.16	0.89 ± 1.63	0.00
SNAQ points	14.78 ± 1.81	16.45 ± 1.48	0.00
Charlson Comorbidity Index	5.02 ± 1.50	4.46 ± 1.20	0.04
Flow speed (m/s)	4.29 ± 0.73	4.50 ± 0.51	0.17
AVA	0.68 ± 0.15	0.76 ± 0.17	0.02
Mean gradient (mmHg)	48.04 ± 14.33	46.62 ± 11.81	0.59
EF (%)	52.00 ± 8.01	51.40 ± 9.43	0.74
EUROScore II	2.98 ± 2.34	3.60 ± 9.87	0.70
CRP (mg/L)	2.24 ± 3.17	4.90 ± 5.00	0.02
Albumin (g/L)	37.89 ± 6.18	37.05 ± 7.64	0.71
Prealbumin (mg/dL)	30.62 ± 7.65	32.53 ± 8.38	0.45

AS—aortic stenosis; AVA—aortic valve area BMI—body mass index; CRP—C reactive protein; EF—ejection fraction; MAMC—mid-arm muscle circumference; CC—calf circumference; HGS—hand grip strength; FFMI—fat-free mass index; SNAQ—simplified nutritional appetite questionnaire; EUROScore II-European System for Cardiac Operative Risk Evaluation.

**Table 6:** Association between nutritional status, appetite, body composition and cardiac parameters.

Parameters A—Elderly with AS; B—Healthy Elderly		F-MNA		7-SGA		SNAQ	
		R	*p*	R	*p*	R	*p*
Aortic valve area (cm^2^)	A	0.23	0.02	0.13	0.20	0.13	0.18
Mean gradient (mm Hg)	A	−0.01	0.89	0.05	0.058	−0.06	0.53
Peak aortic velocity (m/s)	A	0.18	0.13	0.12	0.31	0.11	0.36
%LVEF	A	0.06	0.50	0.10	0.32	−0.10	0.29
EUROScore II	A	−0.15	0.14	−0.27	0.00	−0.04	0.68
Age (year)	A B	−0.23 −0.03	0.01 0.74	−0.29 0.67	0.00 0.13	−0.23 −0.10	0.01 0.36
Age adjusted CCI	A B	−0.24 −0.12	0.01 0.28	−0.17 −0.07	0.08 0.49	−0.10 −0.000	0.31 0.99
% of weight lost in 6 months	A B	−0.43 −0.28	0.00 0.01	−0.33 −0.33	0.00 0.00	−0.04 −0.16	0.66 0.14
Hand grip strength (kg)	A B	0.19 0.08	0.06 0.45	0.23 0.03	0.02 0.75	0.14 −0.16	0.18 0.16
Body mass index (kg/m^2^)	A B	0.38 0.13	0.00 0.25	0.59 0.33	0.00 0.00	0.25 0.16	0.03 0.14
Fat free mass index (kg/m^2^)	A B	0.30 0.04	0.00 0.70	0.39 0.10	0.00 0.36	0.19 −0.08	0.04 0.44
Fat mass index kg/m^2^)	A B	0.21 0.14	0.02 0.20	0.45 0.34	0.00 0.00	0.15 0.24	0.11 0.03
Muscle mass index (kg/m^2^)	A B	0.23 0.04	0.01 0.70	0.33 0.16	0.00 0.16	0.13 −0.09	0.18 0.43
Phase angle 50 kHz (°)	A B	0.18 0.04	0.06 0.72	0.31 0.03	0.00 0.77	0.14 −0.37	0.14 0.00
Mid-arm circumference (cm)	A B	0.42 0.14	0.01 0.25	0.63 0.20	0.01 0.11	0.27 −0.03	0.00 0.81
Calf circumference (cm)	A B	0.35 0.05	0.01 0.68	0.43 0.13	0.00 0.28	0.204 −0.082	0.039 0.517

AS—aortic stenosis; MNA—Mini Nutritional Assessment; SGA—Subjective Global Assessment; SNAQ—Simplified Nutritional Appetite Questionnaire; LVEF—Left Ventricular Ejection Fraction; CCI—Charlson Comorbidity Index.

**Table 7:** Logistic regression analysis: factors related to the deterioration of nutritional status and poor appetite in all subjects.

*n* = 176	7-SGA (<6 Points)		
	OR	% 95 CL	*p*
Aortic stenosis (yes)	3.6	1.30–10.17	0.013
BMI < 24 (kg/m^2^)	13	4.39–40.35	<0.00
Weight lost within 6 months (yes)	4	1.70–9.53	0.001
Age (>75 years)	2.57	1.12–5.87	0.023

SGA—Subjective Global Assessment; BMI—body mass index.
